# Identification of active miRNA and transcription factor regulatory pathways in human obesity-related inflammation

**DOI:** 10.1186/s12859-015-0512-5

**Published:** 2015-03-07

**Authors:** Xi-Mei Zhang, Lin Guo, Mei-Hua Chi, Hong-Mei Sun, Xiao-Wen Chen

**Affiliations:** 1 Department of Histology and Embryology, Harbin Medical University, Harbin, 150081 PR China; 2 Department of Endocrinology and Metabolism, the Second Affiliated Hospital of Harbin Medical University, Harbin, 150081 PR China; 3 Teaching Experiment Center of Morphology, Harbin Medical University, Harbin, 150081 PR China; 4 College of Bioinformatics Science and Technology, Harbin Medical University, Harbin, 150081 PR China

**Keywords:** Obesity, Inflammation, Adipokines

## Abstract

**Background:**

Obesity-induced chronic inflammation plays a fundamental role in the pathogenesis of metabolic syndrome (MS). Recently, a growing body of evidence supports that miRNAs are largely dysregulated in obesity and that specific miRNAs regulate obesity-associated inflammation. We applied an approach aiming to identify active miRNA-TF-gene regulatory pathways in obesity. Firstly, we detected differentially expressed genes (DEGs) and differentially expressed miRNAs (DEmiRs) from mRNA and miRNA expression profiles, respectively. Secondly, by mapping the DEGs and DEmiRs to the curated miRNA-TF-gene regulatory network as active seed nodes and connect them with their immediate neighbors, we obtained the potential active miRNA-TF-gene regulatory subnetwork in obesity. Thirdly, using a Breadth-First-Search (BFS) algorithm, we identified potential active miRNA-TF-gene regulatory pathways in obesity. Finally, through the hypergeometric test, we identified the active miRNA-TF-gene regulatory pathways that were significantly related to obesity.

**Results:**

The potential active pathways with FDR < 0.0005 were considered to be the active miRNA-TF regulatory pathways in obesity. The union of the active pathways is visualized and identical nodes of the active pathways were merged.

**Conclusions:**

We identified 23 active miRNA-TF-gene regulatory pathways that were significantly related to obesity-related inflammation.

**Electronic supplementary material:**

The online version of this article (doi:10.1186/s12859-015-0512-5) contains supplementary material, which is available to authorized users.

## Background

The prevalence of obesity has increased drastically worldwide. Overweight represents major health burdens and is associated with a cluster of metabolic disorders, such as insulin resistence and cardiovascular disease [1]. Obesity is associated with a low-grade inflammation, which influences adipocyte function and may promote type 2 diabetes and MS [2,3].

Adipocytes and infiltrating inflammatory cells located within the adipose tissue (AT) secrete a number of bioactive molecules, such as tumor necrosis fator-α (TNF-α), interleukin (IL)-6 and adiponectin collectively called adipokines. They are labeled as anti-inflammatory or pre-inflammatory adipokines [4,5]. Obesity frequently leads to a dysregulation of adipokine secretion [6,7]. However, the mechanisms controlling adipokines production in obesity are not clear.

In recent years, a lot of evidence has highlighted the modulating roles of miRNAs in inflammatory system [8,9]. MiRNAs may act directly on the target genes or indirectly by first regulating transcription factors (TFs), which then control the expression of genes [9]. The role of miRNAs in adipose inflammation and obesity is still not fully settled. Thus, integrated analysis of transcriptional and post- transcriptional regulation could provide a comprehensive regulatory map for the mechanism study of the AT inflammation in obesity.

In this study, by using hypergeometric test we found 23 active post-transcriptional regulatory pathways in obesity based on obesity-related mRNA and miRNA expression profiles.

## Methods

**Figure 1:**
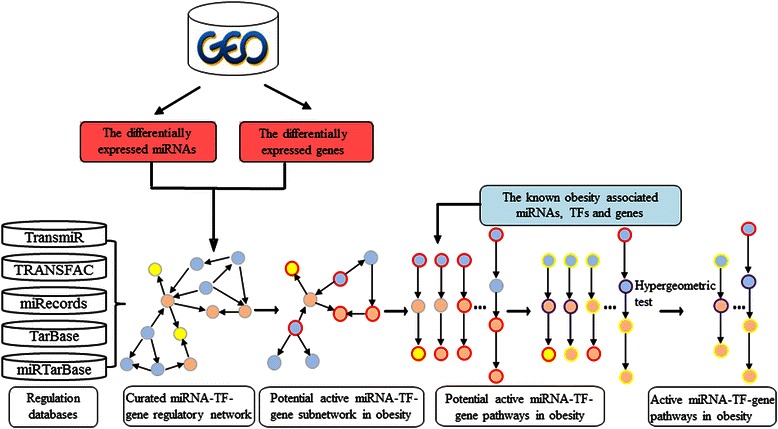
**Flow chart of the proposed approach.** The orange nodes represent miRNAs, the blue nodes represent TFs, and the yellow nodes represent target genes. The nodes with red border indicate the differentially expressed genes and miRNAs, the nodes with purple border indicate the known obesity-associated TFs, adipokines and miRNAs, and the nodes with yellow border indicate the known and differentially expressed genes and miRNAs.

We applied a novel approach to identify active miRNA-TF regulatory pathways in obesity. Firstly, we found differentially expressed genes (DEGs) and differentially expressed miRNAs (DEmiRs) from mRNA and miRNA expression profiles which were derived from the Gene Expression Omnibus (GEO) database [10]. Secondly, we map the DEGs and DEmiRs to the curated miRNA-TF regulatory network as active seed nodes and connect them with their immediate neighbor. In this way, the potential active miRNA-TF regulatory subnetworks in obesity were obtained. Thirdly, based on a BFS algorithm, we identified all of the directed acyclic paths between 0-indegree nodes, where the indegree is 0, and 0-outdegree nodes, where the outdegree is 0. These paths were defined as potential active miRNA-TF regulatory pathways in obesity. Finally, we mapped known obesity-associated miRNAs, TFs and adipokines to potential active pathways. We identified the active miRNA-TF-gene regulatory pathways that were significantly related to obesity based on the hypergeometric test. An overview of the approach is shown in Figure 1.

### Obesity-related mRNA and miRNA expression profiles

The mRNA and miRNA expression profiles (GSE2508 and GSE18470) of human subcutaneous adipose were downloaded from GEO database (Additional file 1: Table S1 and Additional file 2: Table S2).

For mRNA expression profile, 20 lean and 19 obese subjects were hybridized individually to Affymetrix oligonucleotide arrays HG-U95A, B, C, D, and E 6 platforms. In each platform, probe sets were mapped to Entrez Gene IDs. We averaged the expression values of the probe sets which corresponded to the same gene. The differentially expressed genes were detected using Significance Analysis of Microarrays (SAM) approach [11], and false discovery rate (FDR) was used to correct for multiple testing. The genes which were differentially expressed in all of 6 platforms were considered in the analysis.

For miRNA expression profile, we filtered out samples with Type 2 Diabetes Mellitus. Then 6 lean and 13 obese samples were kept for the further analysis. The differentially expressed miRNAs were detected using SAM.

### Curated TF and miRNA regulatory network

We generated TF and miRNA regulatory network by merging miRTarBase,miRecords, TransmiR,TRANSFAC, and TarBase [12-16]. Here, the curated human TF-genes were derived from the TRANSFAC database. The curated TF-miRNA regulations were derived from TransmiR database. The curated human miRNA-gene regulations were obtained from the union of miRecords, TarBase and miRTarBase databases. Within the curated regulatory network, we combined all of the redundant edges into a single edge and deleted all of the self-directed edges from the network [17].

### Known obesity-associated genes and miRNAs

The known obesity-associated genes [18,19] and miRNAs [2,20] are manually collected from published studies.

### Identification of a potential active miRNA –TF regulatory subnetwork in obesity

It was reported that some disease-related key genes could hide among non-DE genes [21,22]. So we hypothesized that the DEGs, DEmiRs, and their immediate neighbors in the constructed miRNA-TF regulatory network potentially induced the pathology of obesity. We generated the potential active miRNA-TF regulatory subnetwork by mapping the DEGs and DEmiRs into the regulatory network as active seed nodes and connecting them with their neighbors [17].

### Identification of potential active miRNA -TF regulatory pathways in obesity

Based on the potential active miRNA-TF-gene regulatory subnetwork, all directed acyclic paths from 0-indegree nodes to 0-outdegree nodes were found. The gene/miRNA with 0-indegree is located upstream of the regulatory pathway. The gene/miRNA with a 0-outdegree is located downstream of the regulatory pathway. And the upstream genes/miRNAs’ activation could cause a cascade effect which results in the regulation of downstream gene/miRNA expression and leads to obesity disease. In this study, we defined potential active regulatory pathways as the directed acyclic paths with more than 2 nodes as, which contained at least one DE node and no more than one non-DE node or nodes without expression values between the two DE nodes [17].

### Evaluation of potential active miRNA-TF-gene regulatory pathways in obesity

Coverage rate (*CR*) of known obesity-associated genes and miRNAs in the potential active pathway is used to measure the strength of the relationships between the potential active pathway and obesity. *CR* was calculated according to

$$ CR=\frac{N_D}{N_T} $$

Where *N*_*D*_ represents the number of known obesity associated genes and miRNAs in the pathway, and *N*_*T*_ represents the total number of genes and miRNAs in the pathway. Then, hypergeometric test were used to evaluate the statistical significance of *CR* value. We further adjusted *p* values for multiple testing using FDR [17].

## Results

### Potential active miRNA -TF regulatory subnetwork in obesity

**Figure 2:**
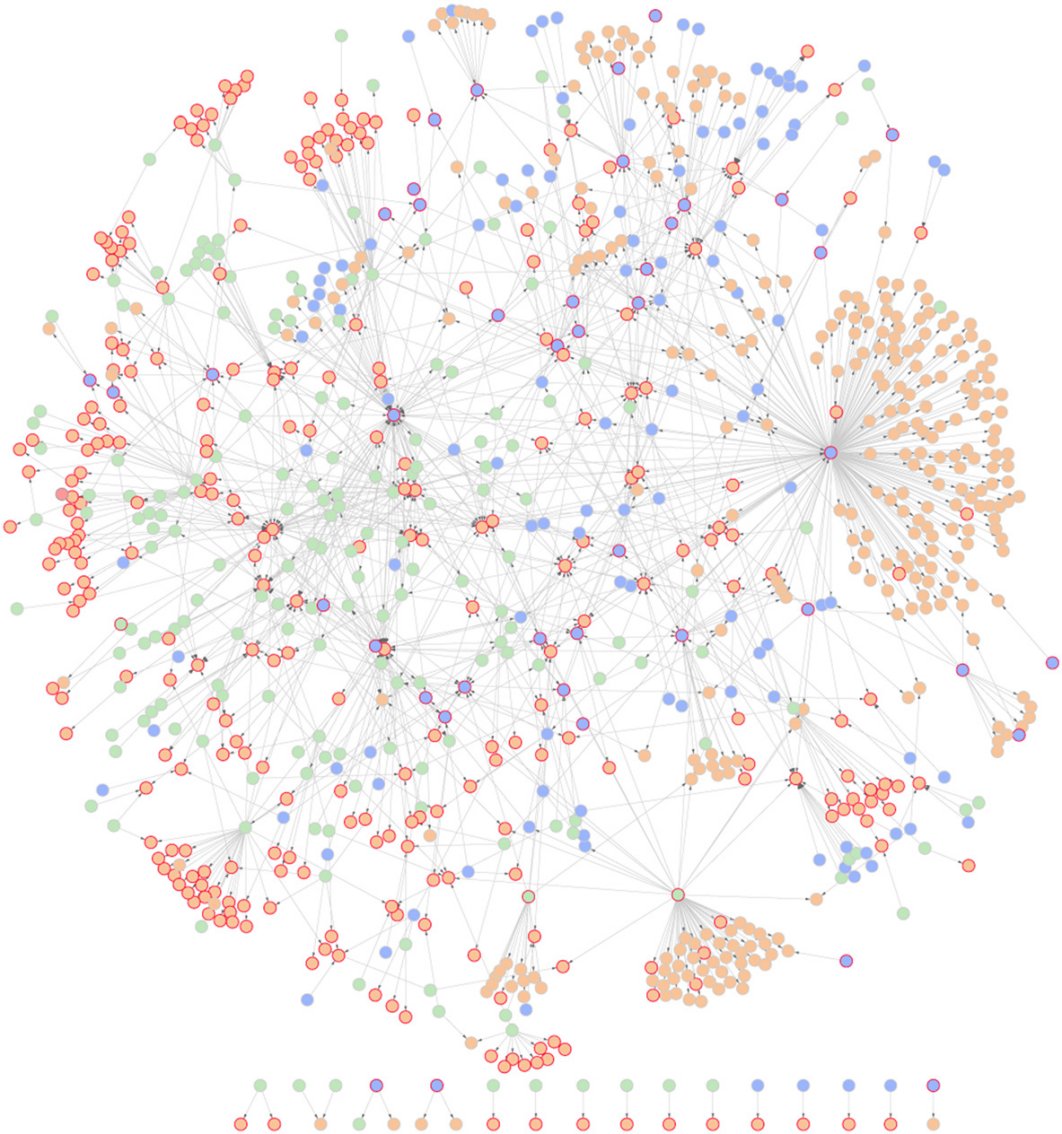
**The potential active miRNA-TF-gene regulatory subnetwork in obesity.** The orange nodes represent miRNAs, the blue nodes represent TFs, and the green nodes represent target genes. The red border indicates the differentially expressed genes and miRNAs.

We identified 1650 DEGs using FDR < 0.01 as threshold and 14 DEmiRs with p-value ≤ 0.05. The transcriptional and post-transcriptional regulations were obtained by integrating from TRANSFAC, TransmiR, miRTarBase, miRecords and TarBase to construct the curated miRNA-TF regulatory network [17]. Then the DEGs and DEmiRs were mapped to the curated miRNA-TF-gene regulatory network as active seeds. We constructed the potential active miRNA-TF-gene regulatory subnetwork by connecting all of the active seeds with their immediate neighbors (Figure 2). Finally, the subnetwork comprised 345 nodes and 1379 edges, in which 1661 genes and 3 miRNAs were differentially expressed.

### The active miRNA-TF-gene regulatory pathways in obesity

We identified all of the directed acyclic paths from 0-indegree nodes to 0-outdegree nodes in the potential active miRNA-TF-gene regulatory subnetwork by BFS approach. As a result, 328800 paths with more than 2 nodes were obtained, which were regarded as the potential active miRNA-TF regulatory pathways in obesity. These pathways contained 568 genes and miRNAs. The length of all of the potential active pathways ranged from 3 to 15, and the average was 11.67.

**Figure 3:**
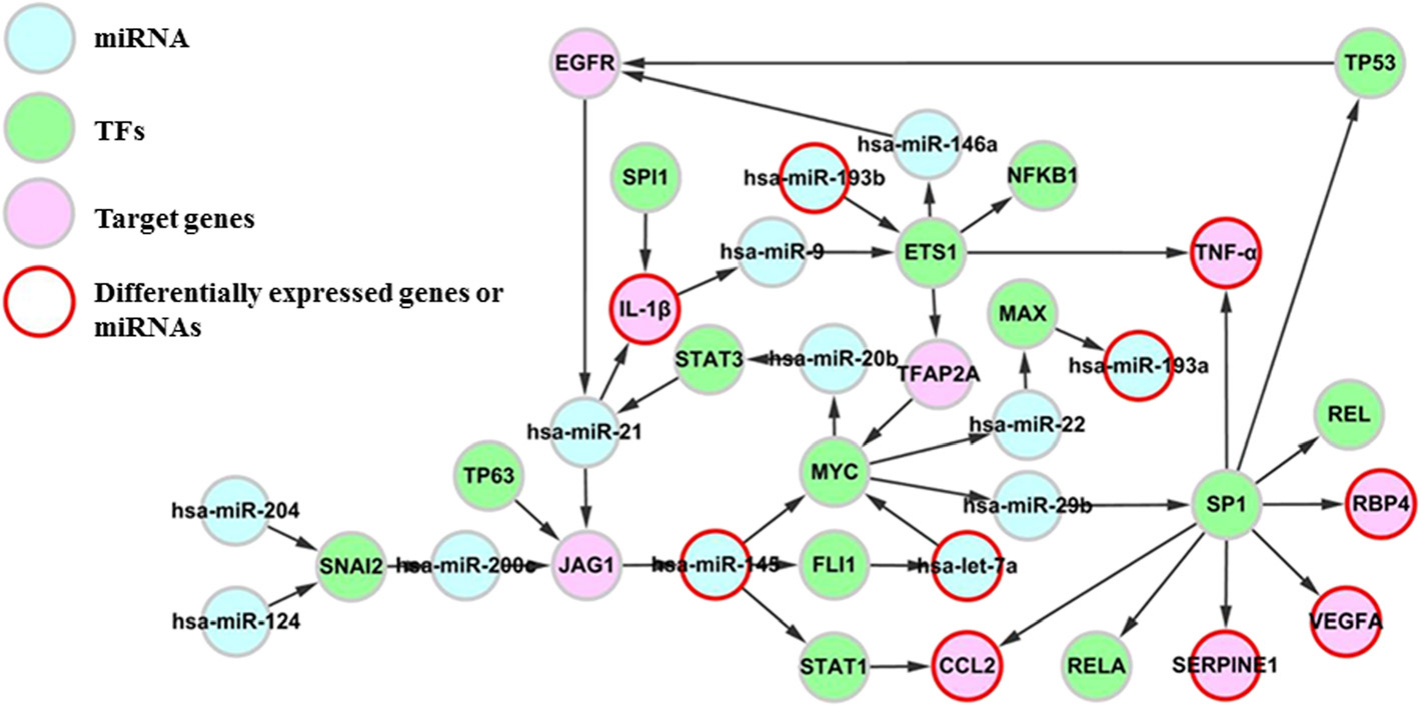
**Union of 23 active miRNA-TF-gene regulatory pathways in obesity.**

Furthermore, we derived 34 known obesity-associated genes, 29 TFs and 11miRNAs to evaluate the association between the identified potential active pathways and obesity. There were 41 obesity-associated genes and miRNAs mapped in the potential active pathways. The coverage rate (*CR*) of the known obesity-associated genes and miRNAs of the potential active pathway was used to measure the strength of the association between the potential active pathway and obesity. Next, we identified the significantly active pathways using a hypergeometric test. The potential active pathways with FDR < 0.0005 were considered to be the active miRNA-TF regulatory pathways in obesity. As a result, we identified 23 active pathways (Table 1). The union of the 23 active pathways is visualized in Figure 3, and identical nodes of the active pathways were merged.

**Table 1:** **Active miRNA-TF-gene regulatory pathways in obesity**

**Active TF-miRNA regulatory pathway**	**Number of known AD genes and miRNAs**	**Pathway length**	**CR value**	**p-value**	**FDR**
hsa-miR-193b → ETS1 → TNF-α	3	3	1	0	0
hsa-miR-193b → ETS1 → NFKB1	3	3	1	0	0
A → FLI1 → hsa-let-7a → MYC → hsa-miR-20b → STAT3 → B → TNF-α	8	15	0.533	8.78E-10	0.000486716
A → MYC → hsa-miR-29b → SP1 → TP53 → EGFR → B → TNF-α	8	15	0.533	8.78E-10	0.000486716
A → FLI1 → hsa-let-7a → MYC → hsa-miR-20b → STAT3 → B → NFKB1	8	15	0.533	8.78E-10	0.000486716
A → MYC → hsa-miR-29b → SP1 → TP53 → EGFR → B → NFKB1	8	15	0.533	8.78E-10	0.000486716
C → hsa-miR-29b → SP1 → TNF	8	15	0.533	8.78E-10	0.000486716
C → hsa-miR-29b → SP1 → RELA	8	15	0.533	8.78E-10	0.000486716
C → hsa-miR-22 → MAX → hsa-miR-193a	8	15	0.533	8.78E-10	0.000486716
C → hsa-miR-29b → SP1 → RBP4	8	15	0.533	8.78E-10	0.000486716
D → FLT1 → hsa-let-7a	8	15	0.533	8.78E-10	0.000486716
C → hsa-miR-29b → SP1 → VEGFA	8	15	0.533	8.78E-10	0.000486716
C → hsa-miR-29b → SP1 → SERPINE1	8	15	0.533	8.78E-10	0.000486716
C → hsa-miR-29b → SP1 → REL	8	15	0.533	8.78E-10	0.000486716
C → hsa-miR-29b → SP1 → CCL2	8	15	0.533	8.78E-10	0.000486716
D → STAT1 → CCL2	8	15	0.533	8.78E-10	0.000486716
C → hsa-miR-29b → SP1 → TP53	8	15	0.533	8.78E-10	0.000486716
E → TNF	8	15	0.533	8.78E-10	0.000486716
E → NFKB1	8	15	0.533	8.78E-10	0.000486716
F → FLI1 → hsa-let-7a → MYC → hsa-miR-20b → STAT3 → G → TNF	8	15	0.533	8.78E-10	0.000486716
F → MYC → hsa-miR-29b → SP1 → TP53 → EGFR → G → TNF	8	15	0.533	8.78E-10	0.000486716
F → FLI1 → hsa-let-7a → MYC → hsa-miR-20b → STAT3 → G → NFKB1	8	15	0.533	8.78E-10	0.000486716
F → MYC → hsa-miR-29b → SP1 → TP53 → EGFR → G → NFKB1	8	15	0.533	8.78E-10	0.000486716

A for hsa-miR-204 → SNAI2 → hsa-miR-200c → JAG1 → hsa-miR-145.

B for hsa-miR-21 → IL-1β → hsa-miR-9 → ETS1.

C for SPI1 → IL1B → hsa-miR-9 → ETS1 → has-miR-146a → EGFR → hsa-miR-21 → JAG1 → .

hsa-miR-145 → FLI1 → hsa-let-7a → MYC.

D for SPI1 → IL1B → hsa-miR-9 → ETS1 → TFAP2A → MYC → hsa-miR-29b → SP1 → TP53 → .

EGFR → hsa-miR-21 → JAG1 → hsa-miR-145.

E for TP63 → JAG1 → hsa-miR-145 → FLI1 → hsa-let-7a → MYC → hsa-miR-29b → SP1 → TP53 → .

EGFR → hsa-miR-21 → IL1B → hsa-miR-9 → ETS1.

F for hsa-miR-124 → SNAI2 → hsa-miR-200c → JAG1 → hsa-miR-145.

G forhsa-miR-21 → IL1B → hsa-miR-9 → ETS1.

## Discussion

Evidence has indicated that miRNAs are largely dysregulated in obesity and that specific miRNAs regulate obesity-associated inflammation [20]. In this study, we proposed a novel approach to identify active miRNA-TF-gene regulatory pathways by integrating obesity-related mRNA and miRNA expression profiles and transcriptional and post-transcriptional regulation. As a result, we identified 23 active miRNA-TF-gene regulatory pathways that were significantly related to obesity.

In these 23 pathways, 6 adipokines including IL-1β, CCL_2_, RBP4, VEGFA,SERPINE4 and TNF-α are involved. IL-1β is regulated by TF SPI1 and has-miR-21. The has-miR-21 is involved in the complex regulation subnet. IL-1β is expressed in and secreted from adipose tissue [23]. IL-1β is a proinflammatory cytokine which has been proposed to play a role in inflammatory pancreatic β-cell destruction leading to type 1diabetes [24]. Obesity is associated with a low-grade inflammatory state in white adipose tissue (WAT). Adipocytes and infiltrating inflammatory cells (primarily macrophages) present within the tissue secrete key inflammatory proteins, such as TNF-a, IL-6, and CCL_2_/monocyte chemoattractant protein, and their gene expression and release are markedly increased in obesity [4,5]. CCL_2_ has been proposed to initiate adipose inflammation by attracting inflammatory cells from the blood stream into WAT and is essential for the development of WAT inflammation [25-27]. The miR-126 reduces the production of CCL_2_ by targeting directly the 3’-UTR of CCL_2_, while miR-193b inhibited indirectly the CCL_2_ production through down regulating the transcription factors of CCL_2_ (RELB, STAT6, and ETS1) [2]. In our detected subnet, TF STAT1 and SP1 directly regulate the expression of CCL_2_, and hsa-miR-145, has- miR-29b through these 2 TFs make regulation on CCL_2_. VEGFA stimulates angiogenesis in adipose tissue,SERPINE4 belongs to pro-inflammatory adipokines, and RBP4 is associated with insulin resistance and visceral fat distribution [18]. They are also regulated by SP1 directly and by has- miR-29b through SP1 indirectly. TNF-α is recognized as a multifunctional cytokine implicated in inflammation, apoptosis and cell survival as well as induction of insulin resistance [28-31]. By use of motif activity response analysis, we were able to dissect pathways linking miRNAs, TFs, and TNF-α. The predicted network comprised several TFs with well-characterized roles in obesity-induced inflammation and three miRNAs that were directly or indirectly linked to TNF-α. For one of the miRNAs, we were able to confirm the regulatory pathways proposed by our model. In particular, we demonstrated a signal circuit from has-miR-193b to TNF-α either via ETS1 as a single TF step. MiRNA-193b may be of particular importance since its expression was associated with TNF-α mRNA expression. Evidence has provided that specific miRNAs regulate the polarization of macrophages and the expression and secretion of adipokines in WAT and a subset of miRNAs could be packaged into adipocyte-derived microvesicles and delivered into blood or neighboring cells, probably acting as inflammatory communicators between adipocytes, macrophages, and distant cells [20]. MiRNAs are secreted into circulation in a stable manner and correlated closely with crucial metabolic parameters, the plasma miRNA profiles could therefore be used as attractive biomarkers for the metabolic syndrome (MS) [20,32,33]. However, whether the signaling circuit established in circulation is also present in adipocytes remains to be determined.

Taken together, our results suggest that specific miRNAs may be important regulators of inflammation in human circulation through their effects on adipokines levels. This may be mediated by indirect effects on TF circuits. miRNAs could therefore constitute novel potential targets for treating obesity comorbidities.

## Conclusions

We identified 23 active miRNA-TF-gene regulatory pathways that were significantly related to obesity-related inflammation.

## Additional files

Additional file 1: Table S1. Obesity-related mRNA expression profiles in adipocytes of obese humans.
Additional file 2: Table S2. Obesity-related miRNA expression profiles in human adipose tissue.
