# Supplementary material for: Identification of active miRNA and transcription factor regulatory pathways in human obesity-related inflammation
Source: BMC Bioinformatics. 2015 Mar 7;16:76. doi: 10.1186/s12859-015-0512-5 (PMC4355475; doi:10.1186/s12859-015-0512-5)
Supplement: Additional file 1: Table S1. — Obesity-related mRNA expression profiles in adipocytes of obese humans. [file 12859_2015_512_MOESM1_ESM.pdf]

Table S1. Obesity-related mRNA expression profiles in adipocytes of obese humans

GEO accession	The body region	Platform	Total samples	Obese samples	Lean samples
GSE2508	Isolated abdominal subcutaneous adipocytes	GPL570	39	19	20

For mRNA expression profile, 20 lean and 19 obese subjects were hybridized individually to Affymetrix oligonucleotide arrays HG-U95A, B, C, D, and E 6 platforms. In each platform, probe sets were mapped to Entrez Gene IDs. We averaged the expression values of the probe sets which corresponded to the same gene. The differentially expressed genes were detected using Significance Analysis of Microarrays (SAM) approach, and false discovery rate (FDR) was used to correct for multiple testing. The genes which were differentially expressed in all of 6 platforms were considered in the analysis.

References

Lee YH, Nair S, Rousseau E, Allison DB, Page GP, Tataranni PA, Bogardus C and Permana PA, *Diabetologia,* 2005, **48**, 1776-1783.
