# Supplementary material for: Identification of active miRNA and transcription factor regulatory pathways in human obesity-related inflammation
Source: BMC Bioinformatics. 2015 Mar 7;16:76. doi: 10.1186/s12859-015-0512-5 (PMC4355475; doi:10.1186/s12859-015-0512-5)
Supplement: Additional file 2: Table S2. — Obesity-related miRNA expression profiles in human adipose tissue. [file 12859_2015_512_MOESM2_ESM.pdf]

Table S2. Obesity-related miRNA expression profiles in human adipose tissue

GEO accession	The body region	Platform	Total samples	Obese without Type 2 Diabetes Mellitus samples	Obese with Type 2 Diabetes Mellitus samples	Lean samples
GSE18470	human subcutaneous adipose tissue	GPL7731	28	13	9	6

For miRNA expression profile, we filtered out samples with Type 2 Diabetes Mellitus. Then 6 lean and 13 obese samples were kept for the further analysis. The differentially expressed miRNAs were detected using SAM.

References

Hummel M, Ferrer A, Rodriguez-Hermosa JI, Ruiz B, Ricart W, Peral B and Fernández-Real JM, *PLoS One*, 2010, **5**, e9022.
